# Antimicrobial Resistance, Genetic Diversity and Multilocus Sequence Typing of *Escherichia coli* from Humans, Retail Chicken and Ground Beef in Egypt

**DOI:** 10.3390/pathogens9050357

**Published:** 2020-05-08

**Authors:** Hazem Ramadan, Charlene R. Jackson, Jonathan G. Frye, Lari M. Hiott, Mohamed Samir, Amal Awad, Tiffanie A. Woodley

**Affiliations:** 1 Bacterial Epidemiology and Antimicrobial Resistance Research Unit, US National Poultry Research Center, USDA-ARS, Athens, GA 30605, USA; jonathan.frye@usda.gov (J.G.F.); lari.hiott@usda.gov (L.M.H.); tiffanie.woodley@usda.gov (T.A.W.); 2 Hygiene and Zoonoses Department, Faculty of Veterinary Medicine, Mansoura University, Mansoura 35516, Egypt; 3 Department of Zoonoses, Faculty of Veterinary Medicine, Zagazig University, Zagazig 44511, Egypt; mohsamir2016@yahoo.com; 4 Bacteriology, Mycology and Immunology Department, Faculty of Veterinary Medicine, Mansoura University, Mansoura 35516, Egypt; amalabdo@mans.edu.eg

**Keywords:** *Escherichia coli*, antimicrobial resistance, humans, retail food, multilocus sequence typing, Egypt

## Abstract

Contamination of retail foods with foodborne pathogens, particularly the antimicrobial resistant ones, poses a persistent threat to human health. There is a dearth of information about the overlapping *Escherichia coli* (*E. coli*) lineages circulating among retail foods and humans in Egypt. This study aimed to determine the clonal diversity of 120 *E. coli* isolates from diarrheic patients (n = 32), retail chicken carcasses (n = 61) and ground beef (n = 27) from Mansoura, Egypt using pulsed-field gel electrophoresis (PFGE) and multilocus sequence typing (MLST). Simpson’s index of diversity was calculated to compare the results of both typing methods. Antimicrobial resistance phenotypes, genotypes and phylogrouping of the isolates were also determined. Higher frequencies of antimicrobial resistance were found among chicken isolates compared to beef and human isolates; regardless of isolate source, the predominant antimicrobial resistances were found against ampicillin (87/120, 72.5%), tetracycline and sulfisoxazole (82/120, 68.3%, each), and streptomycin (79/120, 65.8%). None of the isolates displayed resistance to meropenem. The prevalent genes detected were *tetA* (64.2%), *bla*_TEM_ (62.5%), *sul1* (56.7%), *floR* (53.3%), *sul2* (50%), *strB* (48.3%) and *strA* (47.5%) corresponding with resistance phenotypes. Alarmingly, *bla*_CTX_ was detected in 63.9% (39/61) of chicken isolates. The majority of *E. coli* isolates from humans (90.6%), beef (81.5%) and chicken (70.5%) belonged to commensal phylogroups (A, B1, C). Using PFGE analysis, 16 out of 24 clusters (66.7%) contained isolates from different sources at a similarity level ≥75%. MLST results assigned *E. coli* isolates into 25, 19 and 13 sequence types (STs) from chicken, human and beef isolates, respectively. Six shared STs were identified including ST1011, ST156, ST48, ST224 (chicken and beef), ST10 (human and chicken) and ST226 (human and beef). Simpson’s index of diversity was higher for MLST (0.98) than PFGE (0.94). In conclusion, the existence of common genetic determinants among isolates from retail foods and humans in Egypt as well as the circulation of shared STs indicates a possible epidemiological link with potential zoonotic hazards.

## 1. Introduction

*Escherichia coli (E. coli)*, a member of *Enterobacteriaceae* that inhabits the gastrointestinal tract, is divided into commensal and pathogenic strains that are capable of producing infections in humans and animals [1]. In food-producing animals, both commensal and pathogenic strains of *E. coli* are able to contaminate meat, even in facilities with high standards, with a subsequent potential hazard to humans. The condition is exacerbated if food is contaminated with antimicrobial resistant *E. coli* that could disseminate resistance traits to humans from the food chain and increases the difficulty of treatment of infections in humans [2,3].

The ever-increasing range of antimicrobial resistance in food-producing animals is a global problem. This phenomenon stems from the indiscriminate use of antimicrobials in treatment of animal infections as well as antimicrobial administration in sub-therapeutic doses for prophylaxis and growth promotion [4,5]. In low-resource countries, the problem is complicated with the development of resistances either to antimicrobials that are not licensed for use in animals or to antimicrobials that are prioritized for use in humans only, leading to a subsequent decrease in possible therapeutic choices for the treatment of human illnesses [6,7]. According to World Health Organization (WHO), many European countries and the US banned the use of antimicrobials for animal growth promotion, particularly antimicrobials that are classified as critically important antimicrobials for treatment of human infections [8].

Generally, bacterial mechanisms for antimicrobial resistance are usually associated with either chromosomal mutations and/or acquisition of resistance genes that are commonly linked to mobile genetic elements (MGE) such as plasmids, transposons and integron cassettes [4,9]. To monitor the potential sources of the acquired resistance genes, molecular typing tools are required to identify the common clones of antimicrobial resistant bacteria circulated among different sources. While whole-genome sequencing (WGS) would be the method of choice, it is not always available for use. Of the molecular typing tools, pulsed-field gel electrophoresis (PFGE) and multilocus sequence typing (MLST) are commonly used [10,11,12]. PFGE has been known to have high discriminatory power and is considered the gold standard in *E. coli* sub-typing [13], whereas MLST has the advantage of providing unambiguous sequences from which sequence types (STs) are generated, that could be easily tracked both at national and international levels.

In Egypt, characterization of antimicrobial resistant *E. coli* isolates from food animals has been the subject of many studies [14,15,16,17]; however, most of those studies have focused on conventional phenotypic and genotypic characterization methods without performing a higher resolution molecular typing of such strains. Furthermore, to the best of our knowledge, there have been no reports in Egypt that analyzed *E. coli* from both humans and retail foods, which might articulate the extent of potential transmission sources. Therefore, this study was conducted to determine the genetic diversity of *E. coli* from humans, chicken carcasses and ground beef using PFGE and MLST; antimicrobial resistances for these isolates were also determined.

## 2. Results

### 2.1. Resistance Phenotypes and Genotypes of Human, Chicken and Beef E. coli Isolates

In the present study, antimicrobial susceptibilities of the examined 120 isolates to 14 antimicrobials were identified. Regardless of isolate source, the highest resistance was found against ampicillin (87/120, 72.5%), followed by tetracycline and sulfisoxazole (82/120, 68.3%, each), and streptomycin (79/120, 65.8%). Antimicrobial resistances ranging from 40% to 65% were observed to chloramphenicol (60.8%), quinolones (nalidixic acid, 55%; ciprofloxacin, 43.3%), trimethoprim/sulfamethoxazole (49.2%), ceftriaxone (42.5%), and gentamicin (40.8%). Low frequencies of resistance were detected against cefoxitin (2/120, 1.7%) and amoxicillin/clavulanic acid (4/120, 3.3%); none of the isolates displayed resistance to meropenem. Multidrug resistance (MDR, resistance to at least three antimicrobials in different classes) was identified in 69.1% of the examined isolates (83/120) representing 98.4%, 44.4% and 34.4% of chicken, beef and human isolates, respectively (Table 1). Some of the antimicrobials belonging to the same class exhibited different activity against the examined *E. coli* isolates. For example, cefoxitin and ceftriaxone, both cephalosporins, were inactive against two (1.7%) and 51 (42.5%) *E. coli* isolates, respectively. The resistance phenotype pattern against the majority of examined antimicrobials (11/14, 78.5%) was significantly different (*p*-value < 0.05) with higher frequencies identified among chicken isolates compared to beef and human isolates.

Among the 19 resistance genes examined, *tetA*, *bla*_TEM_, *sul1*, *floR*, *sul2*, *strB* and *strA* were the prevalent genes identified among the examined isolates from retail foods and humans. *bla*_CTX_ was found in 39.2% of the examined isolates (47/120) representing 63.9% (39/61), 18.8% (6/32) and 7.4% (2/27) of chicken, human and beef isolates, respectively. Similar to resistance phenotypes, the distribution of the majority of resistance genes was significantly higher among chicken isolates compared to beef and human isolates (Table 2).

### 2.2. Correlation and Principal Component Analyses of Resistance Phenotypes and Genotypes in E. coli Isolates

The association of resistance phenotypes and genotypes among the examined *E. coli* isolates was determined using correlation analysis. The analysis revealed positive and significant associations between resistance genes, either those conferring resistance to the same antimicrobial class or to different classes, except for *bla*_SHV_ and *bla*_CTX_ that were negatively correlated in a significant manner. Regarding phenotypic/genotypic correlation, in most of the cases, there were significant positive correlations between resistance genes and their corresponding antimicrobials. For instance, *bla*_CTX_, *bla*_TEM_ and *bla*_CMY_ resistance genes were correlated positively and significantly with ceftriaxone. The analysis also showed significant positive correlation between resistance genes and antimicrobials other than the corresponding ones (e.g., *bla*_CTX_ and *bla*_TEM_ genes with sulfisoxazole, streptomycin and tetracycline; *floR* gene with ampicillin, nalidixic acid, sulfisoxazole, streptomycin and tetracycline); however, the only significant negative correlation was detected between *dhfr5* and ciprofloxacin (Figure S1). As shown in the principal component analysis (PCA) plot (Figure S2), the phenotypic as well as genotypic resistance trait could not segregate *E. coli* isolates and the isolates from various sources overlapped largely. The *E. coli* isolates were much closer to each other (less diverse) when analyzing their phenotypic features (Eucladean distance = 2.0) than when analyzing their genotype (Eucladean distance = 2.5).

### 2.3. Phylogrouping of Human, Chicken and Beef E. coli Isolates

The frequency distributions of different *E. coli* phylogroups among the examined *E. coli* isolates are summarized in Table 3. Commensal phylogroups represented by phylogroups A, B1, and C were identified in 90.6% (29/32) of human isolates, 81.5% (22/27) of beef isolates and 70.5% (43/61) of chicken isolates. Meanwhile, pathogenic phylogroups E, B2, and F were detected in chicken isolates (17/61, 27.9%), phylogroups D and E in human isolates (3/32, 9.4%) and phylogroups B2 and E in beef isolates (5/27, 18.5%).

### 2.4. PFGE and MLST Typing of the Examined E. coli Isolates

**Figure 1:**
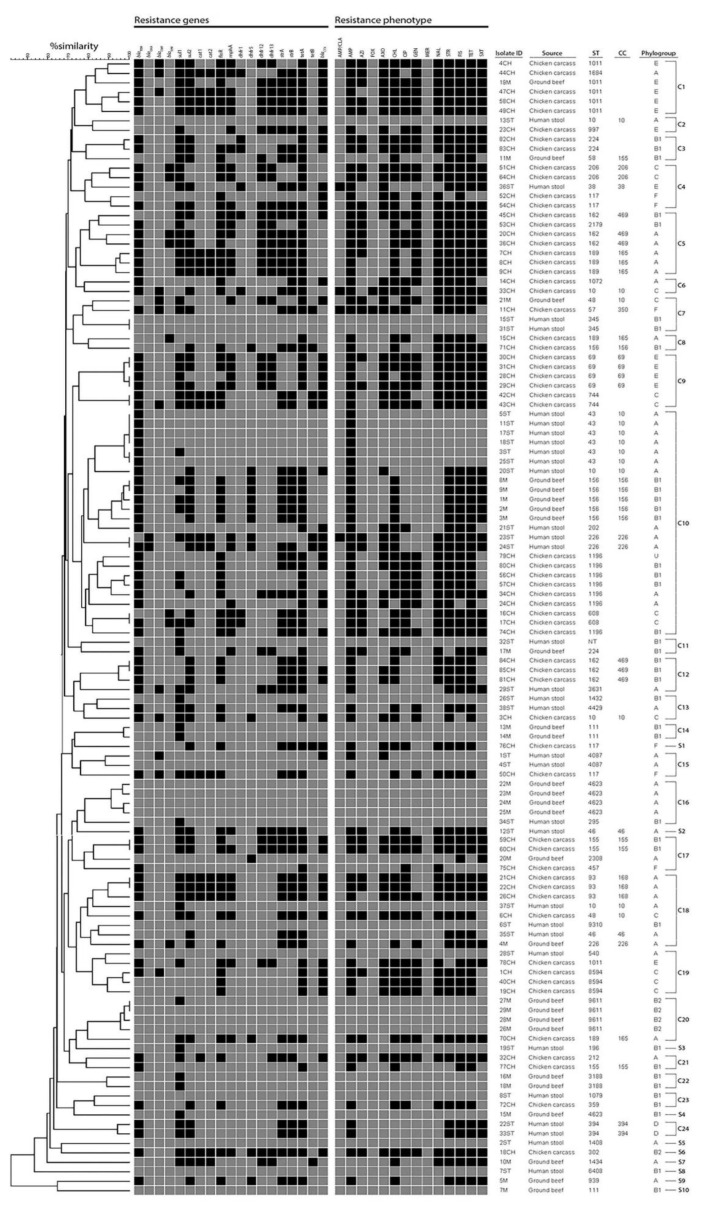
Resistance genotype, phenotype, phylogrouping, pulsed-field gel electrophoresis (PFGE), and multilocus sequence typing (MLST) analyses of *Escherichia coli* isolates from humans, chicken carcasses and ground beef. PFGE clustering of isolates was performed based on band-based Dice coefficient and the unweighted pair group method with arithmetic averages (UPGMA). At a similarity ≥75%, the isolates were clustered into 34 PFGE types including 24 clusters assigned from C1 to C24 and 10 singletons (S1–S10). For resistance genotype and phenotype, black squares indicate the presence of resistance genes and resistance phenotype. Antimicrobials are amoxicillin/clavulanate (AM/CLA), ampicillin (AMP), azithromycin (AZI), cefoxitin (FOX), ceftriaxone (AXO), chloramphenicol (CHL), ciprofloxacin (CIP), gentamicin (GEN), meropenem (MERO), nalidixic acid (NAL), streptomycin (STR), sulfisoxazole (FIS), tetracycline (TET) and trimethoprim/sulphamethoxazole (SXT).

Based on PFGE analysis, clustering of the isolates with ≥75% similarity showed *E. coli* isolates from humans, chicken and beef were distributed into 34 PFGE types including 24 clusters assigned from C1 to C24 and 10 singletons (S1–S10). The 24 clusters encompassed at least two isolates that either belonged to one source (n = 8) or multiple sources (n = 16), known as mixed clusters; meanwhile, a singleton represented one isolate. Of the 16 mixed clusters, only three (C7, C10, C18) clusters contained isolates from all three sources. Meanwhile, the other 13 clusters included isolates from two different sources and were distributed as follows: seven clusters (C2, C4, C12, C13, C15, C19, C23) for isolates from humans and chicken, four clusters (C1, C3, C17, C20) for chicken and beef isolates, and two clusters for human and beef isolates (C11, C16). Cluster C10 was the largest cluster composed of 24 isolates (10 from humans, 9 from chickens and 5 from beef); the human isolate (20ST) harbored similar resistance genes to beef isolates (1M, 2M, 3M, 8M and 9M) except for the presence of *bla*_CTX_ in human isolate and *floR* gene in beef isolates (Figure 1). In some mixed clusters, isolates from different sources were assigned to similar phylogroups (e.g., C1, C3, C10, C11, C18, C23).

**Figure 2:**
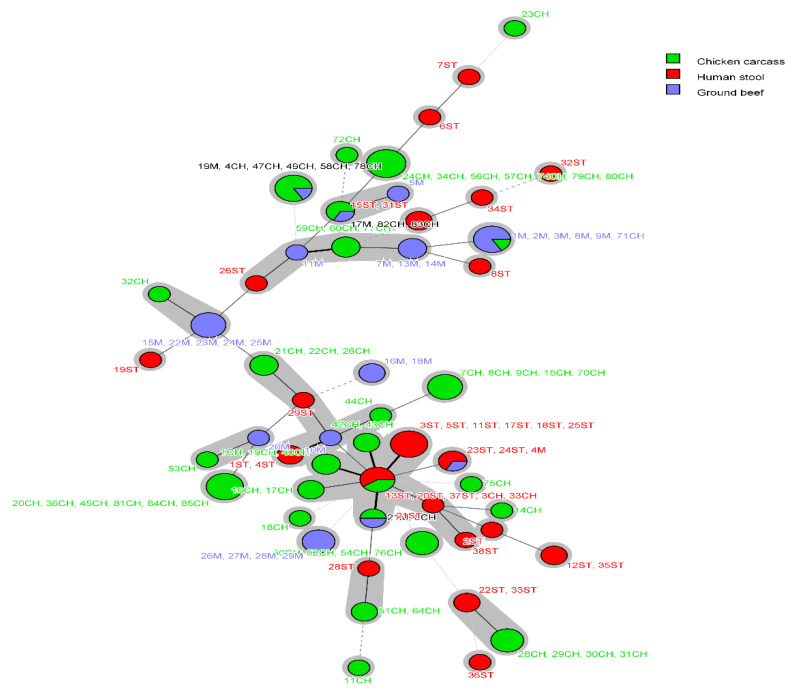
Minimum spanning tree (MST) based on MLST analysis of *E. coli* from humans, chicken carcasses and ground beef. Each circle corresponds to an individual sequence type (ST), and circle size is relevant to the number of isolates assigned to the same ST. The color of the circle denotes the isolate source. Connecting lines (solid and dashed) between circles denote allelic variations between STs, and grey shadowing indicates no more than two different loci between STs.

Using MLST typing, 119 *E. coli* isolates were assigned into 51 different STs, and one isolate from humans was non-typeable. Only 16 STs representing 43.7% (52/119) of isolates were grouped into 13 clonal complexes (CCs). Irrespective of the shared STs among different sources, *E. coli* isolates from humans, chicken carcasses and ground beef were assigned into 19, 25 and 13 STs, respectively (Table S1). Approximately 50% of chicken isolates belonged to six STs: ST1196 (n = 7), ST162 (n = 6), ST189 (n = 5), ST1011 (n = 5), ST69 (n = 4) and ST117 (n = 4). The most prevalent STs among human isolates were ST43 (n = 6) and ST10 (n = 3). Of the 13 STs identified from beef isolates, ST156 (n = 5), ST4623 (n = 5) and ST9611 (n = 4) were the predominant STs that accounted for 52% of beef isolates. Four STs (ST1011, ST156, ST48, ST224) were shared between chicken and beef isolates; whereas, two STs circulated among human and food animal isolates including ST10 (human and chicken isolates) and ST226 (human and beef isolates) (Figure 1 and Figure 2).

Clonal complex (CC)10 was the only CC found in *E. coli* isolates from humans, chicken and beef; it was represented in human isolates by ST43 (n = 6) and ST10 (n = 3), in chicken isolates by ST10 (n = 2) and ST48 (n = 1), and in beef isolates by ST48 (n = 1). Other CCs were identified among isolates from two different sources such as CC155 and CC156 (chicken and beef) and CC226 (human and beef) (Table S1).

Our findings also showed that majority of *E. coli* assigned to similar STs belonged to similar phylogroups. For instance, the overlapping STs (ST1011, ST156, ST48 and ST224) circulating among chicken and beef belonged to phylogroups E, B1, C and B1, respectively, in both sources. Similarly, the human and beef *E. coli* ST226 isolates were assigned to phylogroup A. Moreover, the shared ST10 that was identified in both human and chicken isolates, belonged to phylogroup C in chickens and phylogroup A in humans; both are commensal phylogroups. Likewise, all the identical isolates and sets of isolates that exhibited ≥ 90% similarity within each PFGE cluster were assigned to the same ST.

Simpson’s index of diversity (D) was used to compare between MLST and PFGE as to their ability to discriminate *E. coli* isolates. For both typing methods, the diversity index (discriminatory ability) exceeded 90%, and irrespective of isolate source, MLST had a higher index than PFGE, at approximately 4%. This also remained true when diversity was measured on human and chicken isolates with MLST exhibiting a 5% and 2% increase in discriminatory ability compared to PFGE, respectively, whereas PFGE had a higher discriminatory ability than MLST for beef isolates (Figure S3).

## 3. Discussion

There is a need to monitor the potential sources of human infections using high discriminatory molecular typing methods. This is especially true considering the increase in antimicrobial resistance and the possibility of dissemination of resistance traits, especially those mediated by MGE through the food chain to humans [11,18,19]. In Egypt, the available information about the clonality of antimicrobial resistant *E. coli* from humans and food-producing animals is still minimal. This study provides insight into the overlapping *E. coli* lineages and shared PFGE pulsotypes among isolates from humans and food-producing animals.

In the current study, an overall high prevalence of MDR among the isolates (69.1%) was observed irrespective of their source and when analyzing the entire *E. coli* population within various hosts. The existence of MDR in 98.4% and 44.4% of retail chicken and beef isolates, respectively, with higher resistances against ampicillin, tetracycline, sulfisoxazole and streptomycin, was in concordance with previous studies not only in Egypt [15,16,17], but also worldwide [20,21,22], indicating the selective pressure of these antimicrobials in the treatment of *E. coli* infections in poultry and livestock. Antimicrobial resistances were also detected against chloramphenicol, nalidixic acid, ciprofloxacin, trimethoprim/sulfamethoxazole, ceftriaxone and gentamicin, leaving few therapeutic choices. Fortunately, all the isolates in the current study were sensitive to meropenem, which could be an alternative for the treatment of infections with MDR bacteria. Nonetheless, recent reports from Egypt revealed the existence of carbapenem resistance in *E. coli* isolates from both food producing [14,23] and companion animals [24]. This could provide an evidence about the veterinary use of critically important antimicrobials to humans (e.g., ciprofloxacin, ceftriaxone, cefepime, meropenem, colisitin) in Egypt.

Molecular identification of resistance genes from the examined isolates showed higher frequencies of *tetA*, *bla*_TEM_, *sul1*, *sul2*, *strA*, and *strB* genes that matched higher resistance phenotypes to the corresponding antimicrobials including tetracycline, ampicillin, sulfisoxazole, and streptomycin. Although *bla*_CTX_, which confers resistance to third-generation cephalosporins, is not the predominant β-lactamase in this study, its presence in about 64% of chicken isolates is alarming. This was markedly higher than that previously reported in Egypt from either live birds [25] or chicken meat [14,16,26], with potential hazards to humans, since *bla*_CTX_ is carried on conjugative plasmids and possibly disseminated to humans through food.

The observation that antimicrobials belonging to the same class exhibited variable activity against *E. coli* isolates might indicate discrepancies in the gene content of respective isolates or differences in the use of a particular antimicrobial and thus the selection of resistance. The current study also revealed significant host-dependent differences in phenotypic and genotypic antimicrobial resistance traits. In this, we observed that chicken *E. coli* showed a higher resistance than ground beef isolates, which agrees with a previous study done in Egypt [16]. This could reflect the high usage of antimicrobials for treatment, prophylaxis and growth promotion in chicken than livestock [27].

From correlation analysis, the significant association of resistance genes to the corresponding antimicrobials was expected. However, the existence of significant correlations between resistance genes and other unrelated antimicrobials belonging to different classes could signal co-localization and genetic linkage of these genes either on the chromosome and/or on MGE [28,29]. Despite the differences among the studied sources in *E. coli* isolate profiles, the PCA plot shown in Figure S2 suggests that neither exhibiting a particular resistance phenotype nor harboring certain resistance genes could segregate the sources of *E. coli* into distinct groups. This indicates the high extent of resistance phenotypes and genotypes overlapping among the studied *E. coli* population from humans and food animals.

Molecular subtyping of *E. coli* isolates from humans, chicken and beef was determined using PFGE and MLST. PFGE grouped isolates into 34 distinguishable profiles at a similarity of ≥75%, including 24 clusters and 10 singletons. In 33.3% (8/24) of the clusters, isolates belonging to the same source were grouped together; whereas the clustering of isolates from various sources in mixed clusters (16/24, 67.7%) suggests the existence of shared genetic determinants among these isolates. Likely, the presence of similar phylogroups in some mixed clusters signifies that isolates from different sources might have the same origin at potential animal-to-human transmission.

*E. coli* genotyping using MLST showed a wide diversity of STs; chicken isolates displayed a higher variety of STs followed by human and beef isolates. The predominant STs from chicken isolates differed from the prevalent STs from humans and beef isolates which was in accordance with different studies that showed diversified distribution of *E. coli* lineages among different sources. For instance, in a study performed by Yamaji et al. [11], human and retail meat isolates were assigned into 61 and 95 STs, respectively, and only 12 STs were shared between both isolate sources. In another study from Germany, whole genome sequence analyses revealed the heterogeneous population of *bla*_CMY-2_-producing *E. coli* from humans, livestock animals and food that were assigned into 31, 29 and 20 different STs, respectively [30]. A recent study from Ghana showed the existence of 22 and 13 STs among 34 and 45 *E. coli* isolates from humans and broilers, respectively, with five overlapping STs [31].

Unfortunately, there are limited data about MLST genotyping of *E. coli* from Egypt. A study performed by Fam et al. [32], showed that ST131 was identified in 75% of group B2 clinical *E. coli* isolates from human patients, a pandemic ST that was not identified in the present study. However, other STs that have been previously reported in Egypt were circulating among the current study isolates. For instance, ST1011 that was identified in our study from chicken and beef isolates, was previously recovered from a human patient hospitalized in the intensive care unit of a Cairo City hospital [33]. The predominant ST lineages ST162, ST189, ST1011, and ST117 as well as other STs (ST155, ST93, and ST57) among chicken isolates in this study, have been reported previously in association with avian pathogenic *E. coli* (APEC) and avian fecal *E. coli* (AFEC) from poultry in Egypt [34], indicating the persistent circulation of these clones in poultry in Egypt.

MLST findings also showed the existence of overlapping STs among different sources in the present study with ST1011, ST156, ST48 and ST224 in chicken and beef isolates, ST10 in human and chicken and ST226 in human and beef isolates. Both ST10 and ST48 that belonged to CC10, one of the major CC associated with diarrheagenic *E. coli* infections in humans worldwide [35], were identified in our study from chicken and beef isolates. This suggests the adaptability of certain STs to different hosts with a possible inter-species transmission of these clones.

Simpson’s index of diversity (D) has been used to provide a numerical description, and thus an assessment of the discriminatory ability of bacterial typing methods [36]. In general, both MLST and PFGE were useful approaches in typing *E. coli* and performed well in discriminating *E. coli* isolates as indicated by the above-90% D index upon including or excluding the hosts. This agrees with previous studies of human ESBL producing *E. coli* [37] as well as other bacteria [38,39]. In the current study, we observed an overall 4% increase in the discriminatory ability of MLST compared to that of PFGE. This agrees with a previous study on human *E. coli* isolates, which stated that MLST also exceeds PFGE diversity [37]. The observed difference in D index between both typing schemes is not uncommon and is thought to be partially attributed to the mechanism employed by both methods to pinpoint the genetic change. It is worth mentioning that there is no consensus in the literature regarding the preference of MLST over PFGE since MLST showed less discriminatory power than PFGE in other bacteria [38,40]. Therefore, we do believe that our conclusion is specific to the studied *E. coli* and not universal to other bacterial species.

## 4. Materials and Methods

### 4.1. E. coli Isolates from Humans and Retail Chicken and Beef

One hundred and twenty *E. coli* isolates were randomly chosen for this study from a set of *E. coli* (n = 163) isolated during the period from April to July 2017. Of these, 32 isolates were recovered from human samples and 88 isolates from retail food including 61 isolates from whole chicken carcasses and 27 isolates from ground beef (one isolate per each human and retail food sample). Whole chicken carcasses and ground beef were purchased from retail live chicken shops and supermarkets, respectively in Mansoura, Egypt, and were immediately transported in cooled boxes (4–8 °C) to the laboratory of Hygiene and Zoonoses Department, Faculty of Veterinary Medicine, Mansoura University for bacteriological analysis. Stool samples were provided arbitrarily in sterile specimen cups from diarrheic patients attending Mansoura University Hospitals (MUH), Mansoura, Egypt. Authors were not involved in the sampling process of stool from patients.

Whole chicken carcasses were rinsed thoroughly with tryptic soy broth (TSB; Oxoid, UK) in sterile polyethylene bags and 250 mL of chicken rinsate was incubated at 37 °C for 18 h. Ground beef samples (25 g) were individually homogenized in TSB (225 mL) using a stomacher, and the homogenate was incubated overnight at 37 °C. One gram of each stool sample was suspended in 9 mL of TSB and incubated at 37 °C for 18 h. From the incubated broth of all sample types, a loopful was streaked onto eosin methylene blue (EMB; Oxoid, UK) agar plates and incubated overnight at 37 °C. Typical metallic green sheen colonies were picked from EMB plates and sub-cultured onto blood agar (Oxoid, UK) plates [41]. Presumptive *E. coli* were biochemically confirmed with the automated VITEK2 System (bioMérieux, Durham, NC) according to the manufacturer’s directions. Whole cell template was prepared from each isolate by suspending one colony into 200 μL of sterile nuclease free water, and confirmed as *E. coli* using PCR targeting the *uidA* gene [42].

### 4.2. Antimicrobial Susceptibility Testing of E. coli

Antimicrobial susceptibilities of the recovered *E. coli* isolates from humans and retail food were determined via the microbroth dilution method using the Sensititre^TM^ semi-automated susceptibility system (TREK Diagnostic Systems, Inc., Westlake, OH, USA) and the National Antimicrobial Resistance Monitoring System (NARMS) plate CMV3AGNF, according to Clinical and Laboratory Standards Institute (CLSI) guidelines [43]. Minimum inhibitory concentrations (MICs) were determined based on the breakpoints listed in CLSI standards as follows: ampicillin (≥32 μg/mL), amoxicillin/clavulanic acid (≥32/16 μg/mL), cefoxitin (≥32 μg/mL), ceftriaxone (≥4 μg/mL), meropenem (≥4 μg/mL), chloramphenicol (≥32 μg/mL), azithromycin (≥32 μg/mL), ciprofloxacin (≥4 μg/mL), nalidixic acid (≥32 μg/mL), sulfisoxazole (≥512 μg/mL), tetracycline (≥16 μg/mL), trimethoprim/sulfamethoxazole (≥4/76 μg/mL), and gentamicin (≥16 μg/mL). For streptomycin, which does not have a breakpoint determined by CLSI, result interpretation was determined according to the NARMS breakpoint (≥64 μg/mL). Reference strains, *E*. *coli* ATCC 25922, *Pseudomonas aeruginosa* ATCC 27853, *Staphylococcus aureus* ATCC 29213 and *Enterococcus faecalis* ATCC 29212, were used for quality control.

### 4.3. Resistance Genotypes and E. coli Phylogrouping

PCR with whole cell templates of all 120 *E. coli* isolates was used to screen for the presence of genes encoding resistance to β-lactams (*bla*_CTX_, *bla*_TEM_, *bla*_CMY_, *bla*_SHV_, *bla*_OXA_), chloramphenicol (*cat1*, *cat2*, *floR*), tetracycline (*tetA*, *tetB*), sulfisoxazole (*sul1*, *sul2*), streptomycin (*strA*, *strB*), trimethoprim/sulfamethoxazole (*dhfr1*, *dhfr5*, *dhfr12*, *dhfr13*), and azithromycin (*mphA*) as previously described [19,44,45]. PCR assay for each resistance gene was performed in 25 μL reaction mixture containing 2.5 μL of each 10 pmole primer (Eurofins Genomics, Huntsville, AL, USA), 1.5 μL MgCl_2_ (30 mM), 0.5 μL dNTP (2 mM) (Roche, Indianapolis, IN, USA), 0.25 μL Taq polymerase (5 U/μL) (Roche, Indianapolis, IN, USA) and 3 μL of DNA template. Positive and negative controls were included in each PCR run. *E. coli* phylogrouping into one of 8 phylogroups: A, B1, C, B2, D, F, E, and *Escherichia* cryptic clade I, was performed according to Clermont et al. [46].

### 4.4. Pulsed-Field Gel Electrophoresis (PFGE) Analysis

PFGE was performed to determine the genetic relatedness among *E. coli* isolates from humans, retail chicken and beef according to PulseNet [13]. Briefly, *E. coli* genomic DNA was embedded in 1% Seakem Gold agarose (BioWhittaker Molecular Applications, Rockland, ME, USA) and agarose plugs were digested with 10 U of *Xba*I (Roche Molecular Biochemicals, Indianapolis, IN, USA). Digested fragments were electrophoresed using the CHEF-DRII system (Bio-Rad, Hercules, California, CA, USA) at 6 V with a ramped pulse time of 2.16–54.17 s for 19 h at 14 °C. Gel images were imported into BioNumerics software (Applied Maths, Austin, TX, USA, version 7.6) and the dendrogram was generated using the band-based Dice coefficient and the unweighted pair group method with arithmetic averages (UPGMA).

### 4.5. Multilocus Sequence Typing (MLST)

PCR amplification of seven housekeeping genes (*adk*, *fumC*, *gyrB*, *icd*, *mdh*, *purA*, *recA*) was performed according to Wirth et al. [47] to identify the sequence types (STs) of the examined *E. coli* isolates. The amplified products for each gene were purified with a QIAquick PCR Purification Kit (Qiagen, Inc., Valencia, CA, USA) according to the manufacturer’s instructions. Sanger sequencing of the purified amplicons using ABI 3730XL sequencer (Applied Biosystems Inc., Foster City, CA, USA) was performed at the USDA Genomics and Bioinformatics Research Unit, Stoneville, MS, USA. Sequences were imported into BioNumerics software (Applied Maths, Austin, TX, USA, version 7.6), assembled and trimmed; the allelic profiles of the seven housekeeping genes, STs and clonal complexes (CCs) were obtained from the *E. coli* MLST database (https://pubmlst.org/bigsdb?db=pubmlst_mlst_seqdef). Minimum spanning tree (MST) was generated using BioNumerics software (Applied Maths, Austin, TX, USA, version 7.6).

### 4.6. Statistical Analyses

To compare the discriminatory ability of PFGE and MLST of *E. coli* typing, Simpson’s index of diversity (D) [48] was calculated using the following formula 1-D. In this equation, D = $\sum \frac{n(n-1)}{N(N-1)}$, where ∑ = summation, n = number of bacterial isolates showing a particular PFGE or MLST type and N = total number of bacterial strains in the respective source. To determine if the differences in the frequency of resistance phenotype, resistance genes or phylogroups in *E. coli* was significant among investigated hosts, these frequencies were used as inputs to create contingency tables and the significance was determined by X^2^ test (Chi-square or Fisher exact test, whenever needed), with a cutoff level for *p*-value equal to 0.05.

To determine the extent of correlation between the antibiotic-resistance phenotype and the presence of resistance genes, the profile of resistance against an antibiotic and the presence of resistance genes were entered as 1, whereas sensitivity to an antibiotic and absence of resistance genes were scored as 0. To calculate the correlation, the “cor” and “cor.test” functions from the software “R, v. 3.6.1” were utilized. Significant correlation was visualized using “corrplot” function from the “corrplot” package in the software “R, v. 3.6.1” [49]. To visualize the overlap between human and retail food isolates, binary data representing the isolates phenotypic and genotypic profiles were used to generate a principal component analysis (PCA) plot based on the Euclidean distances among isolate pairs. This analysis was done using PC-ORD Software (v. 5, MjM Software, Gleneden Beach, OR, USA).

## 5. Conclusions

This study determined the existence of shared antimicrobial resistances, phylogroups, PFGE types and ST clones among *E. coli* isolates from retail foods and humans. While the knowledge gained from this study complements previous reports in Egypt, the provided STs would be valuable for epidemiological surveillances of foodborne outbreaks in Egypt, and possibly other countries by highlighting potential transmission sources. Further study is planned to apply whole-genome analyses for the shared ST lineages from retail food and humans, which will improve our knowledge about the possible mechanisms of potential animal-to-human transmission and help to set up control interventions.

## Figures and Tables

**Table 1:** Frequencies of antimicrobial resistance phenotypes among *E. coli* isolates from humans, chicken carcasses and ground beef in Egypt.

Antimicrobial Agent	Antimicrobial Class	Total (n = 120) No. Resistant (%)	Chicken (n = 61) No. Resistant (%)	Beef (n = 27) No. Resistant (%)	Human (n = 32) No. Resistant (%)	*p*-Value
AM/CLA	β-Lactam/β-Lactamase inhibitor	4 (3.3)	2 (3.3)	0	2 (6.3)	0.540000
AMP	Penicillins	87 (72.5)	60 (98.4)	10 (37)	17 (53.1)	<0.0001
AXO	Cephalosporins	51 (42.5)	43 (70.5)	2 (7.4)	6 (18.8)	<0.0001
FOX		2 (1.7)	2 (3.3)	0	0	0.720000
NAL	Quinolones	66 (55)	59 (96.7)	4 (14.8)	3 (9.4)	<0.0001
CIP		52 (43.3)	47 (77)	3 (11.1)	2 (6.3)	<0.0001
STR	Aminoglycosides	79 (65.8)	57 (93.4)	11 (40.7)	11 (34.4)	<0.0001
GEN		49 (40.8)	45 (73.8)	3 (11.1)	1 (3.1)	<0.0001
SXT	Folate pathway inhibitors	59 (49.2)	39 (63.9)	12 (44.4)	8 (25)	0.0014
FIS		82 (68.3)	58 (95.1)	13 (48.1)	11 (34.4)	<0.0001
AZI	Macrolides	37 (30.8)	31 (50.8)	3 (11.1)	3 (9.4)	<0.0001
TET	Tetracyclines	82 (68.3)	59 (96.7)	12 (44.4)	11 (34.4)	<0.0001
CHL	Phenicols	73 (60.8)	57 (93.4)	11 (40.7)	5 (15.6)	<0.0001
MERO	Carbapenems	0	0	0	0	NA
Multidrug resistance (MDR) No. of isolates (%)		83 (69.1)	60 (98.4)	12 (44.4)	11 (34.4)	<0.0001

Amoxicillin/clavulanate, AM/CLA; Ampicillin, AMP; Azithromycin, AZI; Cefoxitin, FOX; Ceftriaxone, AXO; Chloramphenicol, CHL; Ciprofloxacin, CIP; Gentamicin, GEN; Meropenem, MERO; Nalidixic acid, NAL: Streptomycin, STR; Sulfisoxazole, FIS; Tetracycline, TET; Trimethoprim/sulphamethoxazole, SXT. NA; Not applicable.

**Table 2:** Frequencies of resistance genes among *E. coli* isolates from humans, chicken carcasses and ground beef in Egypt.

Resistance Gene	Total (n = 120) No. (%)	Chicken (n = 61) No. (%)	Beef (n = 27) No. (%)	Human (n = 32) No. (%)	*p*-Value
*bla*_TEM_	75 (62.5)	51 (83.6)	9 (33.3)	15 (46.9)	<0.0001
*bla*_CTX_	47 (39.2)	39 (63.9)	2 (7.4)	6 (18.8)	<0.0001
*bla*_CMY_	12 (10)	9 (14.8)	1 (3.7)	2 (6.3)	0.1998
*bla*_SHV_	8 (6.7)	7 (11.5)	1 (3.7)	0	0.0849
*bla*_OXA_	2 (1.7)	0	0	2 (6.3)	0.061
*cat1*	18 (15)	15 (24.6)	1 (3.7)	2 (6.3)	0.011
*cat2*	17 (14.2)	14 (23)	1 (3.7)	2 (6.3)	0.0188
*floR*	64 (53.3)	53 (86.9)	9 (33.3)	2 (6.3)	<0.0001
*tetA*	77 (64.2)	57 (93.4)	11 (40.7)	9 (28.1)	<0.0001
*tetB*	8 (6.7)	5 (8.2)	1 (3.7)	2 (6.3)	0.7337
*sul1*	68 (56.7)	41 (67.2)	14 (51.9)	13 (40.6)	0.0413
*sul2*	60 (50)	40 (65.6)	10 (37)	10 (31.3)	0.0022
*strA*	57 (47.5)	39 (63.9)	9 (33.3)	9 (28.1)	0.0011
*strB*	58 (48.3)	40 (65.6)	9 (33.3)	9 (28.1)	0.0006
*dhfr1*	8 (6.7)	7 (11.5)	0(0)	1 (3.1)	0.0889
*dhfr5*	14 (11.7)	4 (6.6)	7 (25.9)	3 (9.4)	0.0297
*dhfr12*	31 (25.8)	24 (39.3)	5 (18.5)	2 (6.3)	0.0015
*dhfr13*	31 (25.8)	25 (41)	4 (14.8)	2 (6.3)	0.0004
*mphA*	37 (30.8)	32 (52.5)	2 (7.4)	3 (9.4)	<0.0001

**Table 3:** Frequencies of phylogroups among *E. coli* isolates from humans, chicken carcasses and ground beef in Egypt.

Phylogroup	Total (n = 120) No. (%)	Chicken (n = 61) No. (%)	Beef (n = 27) No. (%)	Human (n = 32) No. (%)	*p*-Value
A	43 (35.8)	15 (24.6)	8 (29.6)	20 (62.5)	0.0011
B1	38 (31.7)	16 (26.2)	13 (48.1)	9 (28.1)	0.1103
C	13 (10.8)	12 (19.7)	1 (3.7)	0	0.006
B2	5 (4.2)	1 (1.6)	4 (14.8)	0	0.01
D	2 (1.7)	0	0	2 (6.3)	0.1186
E	12 (10)	10 (16.4)	1 (3.7)	1 (3.1)	0.0596
F	6 (5)	6 (9.8)	0	0	0.05
Clade I	0	0	0	0	NA
U *	1 (0.83)	1 (1.6)	0	0	0.99

* One chicken isolate could not be assigned a phylogroup (untypeable; U). NA. Not available.

## Supplementary Materials

The following are available online at https://www.mdpi.com/2076-0817/9/5/357/s1, Figure S1: Correlation matrix showing the significant (*p*-value < 0.05) correlation (r) between resistance phenotypes and genotypes among the examined *E. coli* isolates, Figure S2: Principle component analysis (PCA) of resistance phenotypes and genotypes among the examined *E. coli* isolates, Figure S3: Discriminatory power of PFGE and MLST as measured by Simpson’s index of diversity (D) among the examined *E. coli* isolates, Table S1: Distribution of specific sequence types (STs), shared STs and clonal complexes (CCs) among *E. coli* isolates from humans, chicken carcasses and ground beef in Egypt.
