# Supplementary material for: Antimicrobial Resistance, Genetic Diversity and Multilocus Sequence Typing of Escherichia coli from Humans, Retail Chicken and Ground Beef in Egypt
Source: Pathogens. 2020 May 8;9(5):357. doi: 10.3390/pathogens9050357 (PMC7281645; doi:10.3390/pathogens9050357)
Supplement: Supplementary file 1 [file pathogens-09-00357-s001.pdf]

Supplementary data

Table S1. Distribution of specific sequence types (STs), shared STs and clonal complexes (CCs) among *E. coli* isolates from humans, chicken carcasses and ground beef in Egypt.

Source	Specific STs<br>ST (no.)	Shared STs<br>ST (no.)	Shared CCs
Chicken	8594 (3), 189 (5), 57 (1), 1072 (1), 608 (2), 302 (1), 162 (6), 93 (3), 997 (1), 1196 (7), 69 (4), 212 (1), 744 (2), 1684 (1), 117 (4), 206 (2), 2179 (1), 155 (3), 359 (1), 457 (1)	ST10 (2), ST48 (1), ST1011 (5), ST156 (1), ST224 (2)	CC10 , CC155 , CC156
Human	43 (6), 4087 (2), 1408 (1), 9310 (1), 6408 (1), 1079 (1), 46 (2), 345 (2), 196 (1), 202 (1), 394 (2), 1432 (1), 540 (1), 3631 (1), 295 (1), 38 (1), 4429 (1)	ST10 (3), ST226 (2)	CC10 , CC226
Beef	939 (1), 111 (3), 1434 (1), 58 (1), 4623 (5), 3188 (2), 2308 (1), 9611 (4)	ST226 (1), ST48 (1), ST1011 (1), ST156 (5), ST224 (1)	CC10 , CC155 , CC156 , CC226

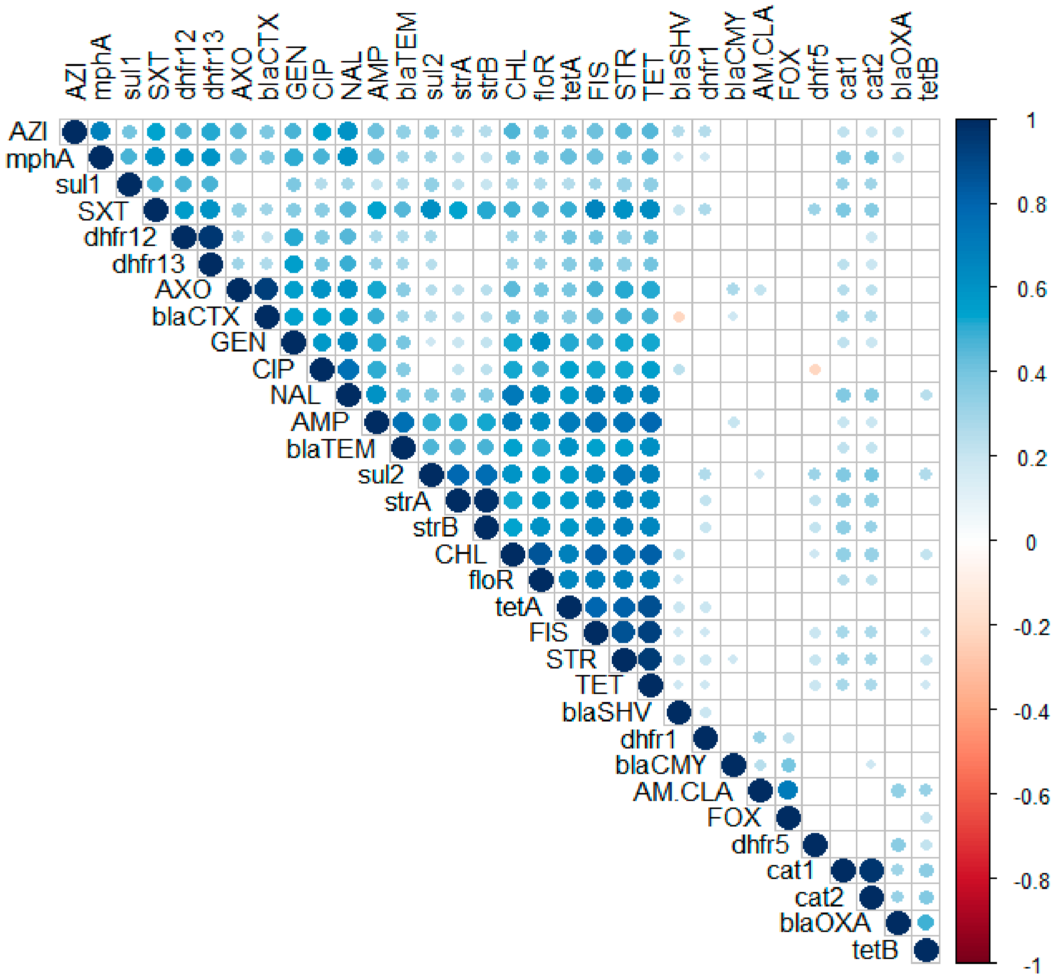

Figure S1. Correlation matrix showing the significant (*P*-value < 0.05) correlation (r) between resistance phenotypes and genotypes among the examined *E. coli* isolates. The size and strength of the colors correspond to the numerical value of the correlation coefficient. Blue color indicate positive correlation and red color indicates negative correlation. The blank cells indicate non-significant correlation.

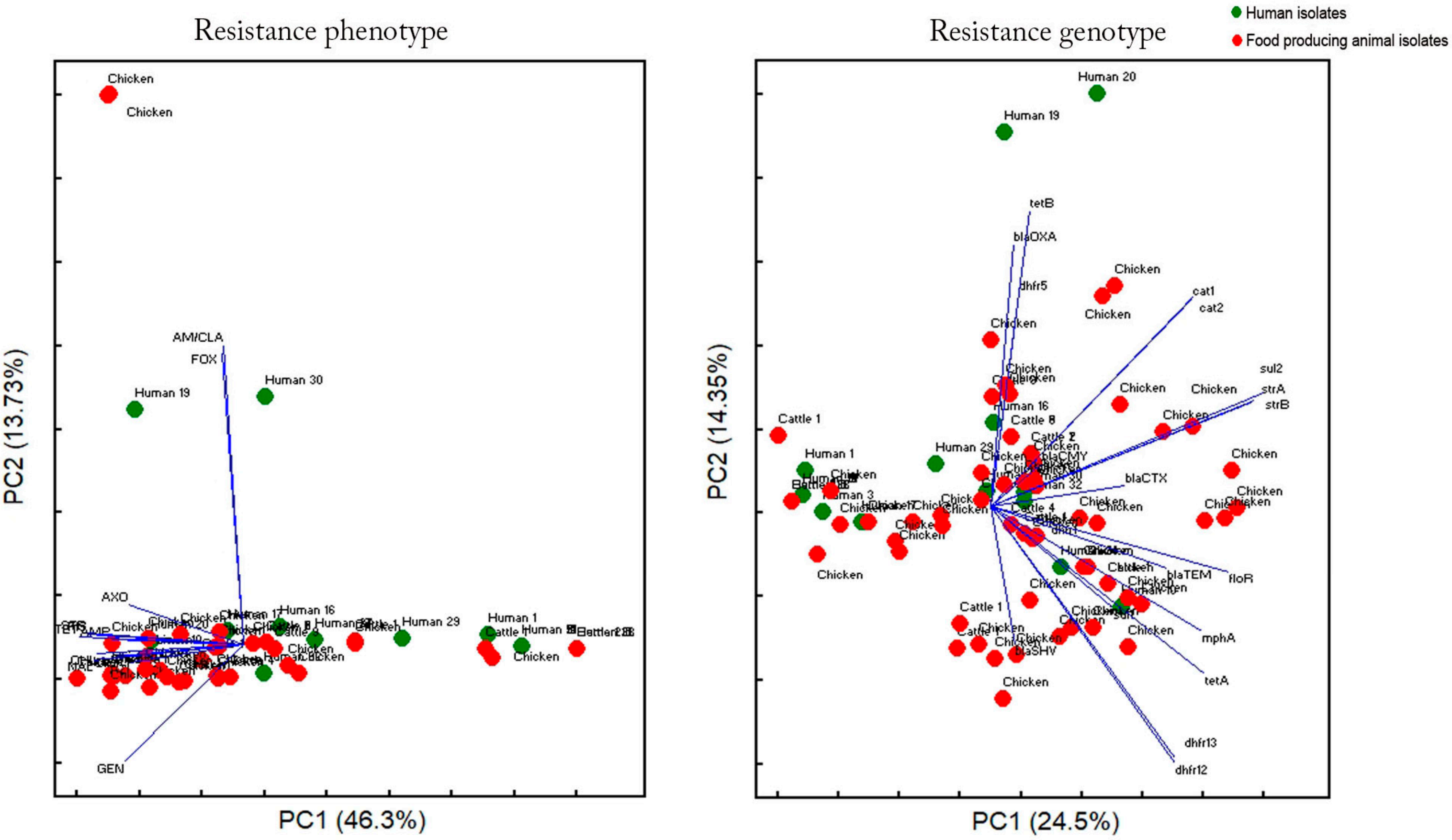

Figure S2. Principle component analysis (PCA) showing the clustering pattern of human and retail food isolates based on the frequency distribution of resistance phenotypes and genotypes.

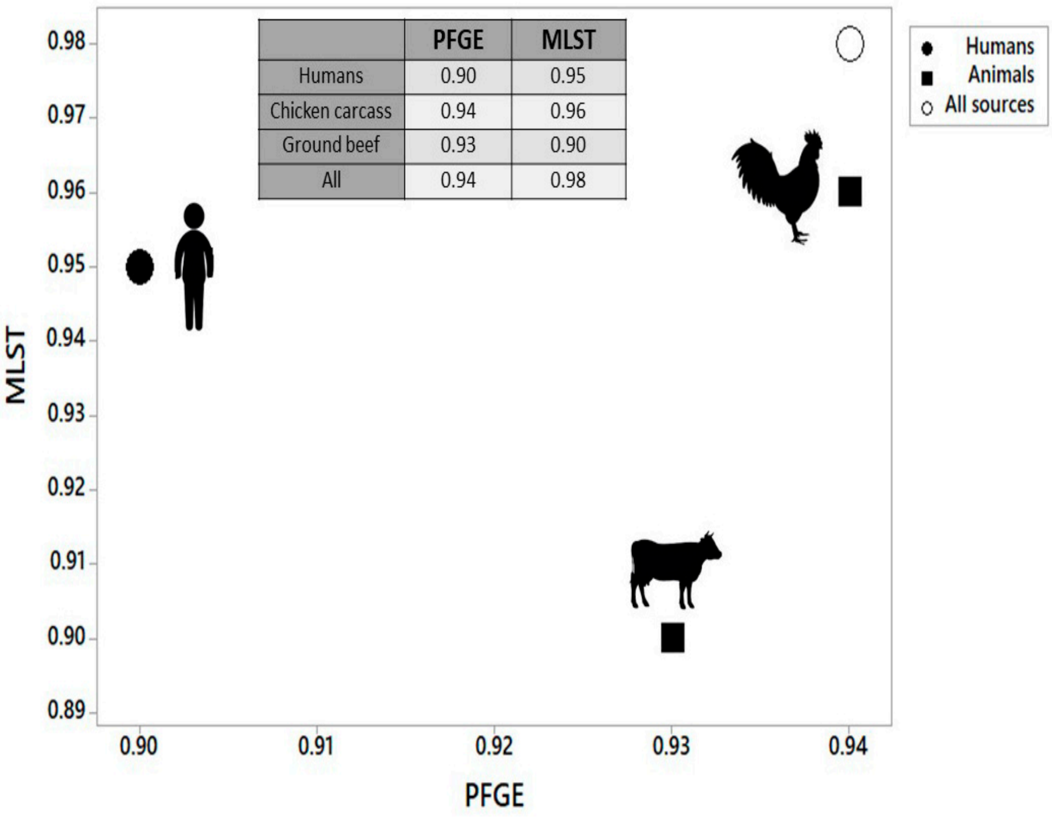

Figure S3. Discriminatory power of PFGE (shown on *X*-axis) and MLST (shown on *Y*-axis) as measured by Simpson's index of diversity (D) among the examined *E. coli* isolates.
